# Supplementary material for: Conservation of Three-Dimensional Structure of Lepidoptera and Trichoptera L-Fibroins for 290 Million Years
Source: Molecules. 2022 Sep 13;27(18):5945. doi: 10.3390/molecules27185945 (PMC9504780; doi:10.3390/molecules27185945)
Supplement: Supplementary file 1 [file molecules-27-05945-s001.zip › molecules-1882336-supplementary.pdf]

```
P21828      FIBL_BOMMO             1  ----MKPIFL--VLLVATSAYAAP-SVTINQYSDNEIPRDIDDGKAS--SVISRAWD-YV   50
Q26427      FIBL_GALME             1  ----MLPFVL--VLLVATSALAAP-SVVISQDNINNIAPRVGNGRPISSALIDRAFE-IV   52
Q9BLL5      Q9BLL5_PAPXU           1  ----MLPITL--ILLVVTSAFAAP-SVTVTQYNANDIPPVPDNGKPSSSI--ENAFDVLS   51
A0A6J2JLC8  A0A6J2JLC8_BOMMA       1  ----MKPIFL--VLLVATSAYAAP-SVTINQYSDNEIPRDIDDGKAS--SVISRAWD-YV   50
A0A7I0ZNV9  A0A7I0ZNV9_DANPL       1  MTCTMLPFVL--VFLFAQSTFALPTA-LVNFVDVNEARPVIDNGQLVSRALIDKVFE-LI   56
Q9BLL7      Q9BLL7_9NEOP           1  ---MMRPIVL--VLLFATSALAAP-SVLLKQYSENEVAPTKDNGKQVSSYLTDRTFD-LF   53
A0A891XIJ7  A0A891XIJ7_TINBI       1  ----MLRFAL--VLFAAQCAFAAP-QVQIIAQNIDEVPRPRDNGRQVNSYLGTGLFELLD   53
Q14UU5      Q14UU5_YPOEV           1  ----MLPLVL--VLLVAQSALSAP-SVSVNQVAYNQAEGPRDNGNLINSYVTDAVFG-LL   52
A0A0N1PIW2  A0A0N1PIW2_PAPMA       1  ----MLPITL--ILLVVTSAFAAP-SVTVTQYNANDIPPVPDNGKPSSSI--ENAFSVLN   51
A0A3S2NFE9  A0A3S2NFE9_CHISP       1  ----MLPLVL--VSLLVSGALAAAVPVTLTQYSINEIPPLNENGRFISSALTRNALD-NV   53
A0A2A4JXS4  A0A2A4JXS4_HELVI       1  ----MLPIVL--VLLVATSALAAP-GVNLSIRNVNEVVPPRHNGRAVSAFLTNQAFE-NI   52
A0A821XWV1  A0A821XWV1_9NEOP       1  ----MLPFVL--VLLAAQSAFAAPSNAAVTFYNTNEVGSVPDNGGLVNSYIVSGPVD-YL   53
A0A8B8HT70  A0A8B8HT70_9NEOP       1  ----MLPIVL--VLFVAQSAFAAPSVVGVQFKNFNEVAPVSDNGKLVSSYITDGSIA-YI   53
D0EWD8      D0EWD8_CORCP           1  ----MLPFVL--VLLVASSALAAP-SVSITQDNINNIAPVASNGRAVSSYLTDRAFE-IV   52
J9XSZ1      J9XSZ1_HARDE           1  ----MLPFVL--VSLFVSGALAVP-VVNVNQYSINEVAPVGDNGRLVSSFLTDRSFE-AV   52
V5UTC1      V5UTC1_OSTFU           1  ----MLPFVL--VSLLVSGAFALP-AVSVSQYNYNEIAPVADNGKLVSSYLSSNAFD-LV   52
A0A7E5W904  A0A7E5W904_TRINI       1  ----MLPVVL--LLL-AASALANP-FGRVNQVSYDDVG---------SNSVIEGILD-SV   42
A0A6J1NAW1  A0A6J1NAW1_BICAN       1  ----MLPLIL--VLFLAQNALSLPYT-NVNFYNIENAPSIPDNGQLINSNVTTGALE-WI   52
A0A5B2VHG3  A0A5B2VHG3_9BACT       1  ----MKPIFL--VLLVATSAYAAP-SVTINQYSDNEIPRDIDDGKAS--SVISRAWD-YV   50
A0A835GRS5  A0A835GRS5_SPOEX       1  ----MLPVVL--VLLAATSALAAP-GAYLNIISIDEAIPPKDSGRAVSAFRIDQATE-IA   52
D7PBV9      D7PBV9_9NEOP           1  ----MIPIIVLSVLLAAQGAYG----ASVNFVPIEDVPIRYEFGNTINRNVLDNAFQ-LV   51
A0A7U3TKS2  A0A7U3TKS2_PLUXY       1  ----MLPIVL--VLLVAQSAFGAP-AVQLSLANFNEVAPSKDNGRLVNRHVSDGAFD-LI   52
Limnephilus_decipienns             1  ----MALSLLIGALLAIQGASFVA-------------------SSHISASLLEGTWD-LV   36
Limnephilus_lunatus                1  ----MALSLLIGALLAIQGASFVA-------------------SSHISASLLERTWD-LV   36
Limnephilus_rhombicus              1  ----MALSLLIGALLAIQGASFVA-------------------SSSISASLLEATWD-LV   36
Limnephilus_marmoratus             1  ----MALSLLIGALLAIQGASFVA-------------------SSHVSSSLLEATWD-LV   36
Hesperophylax_occidentalis         1  ----MALSLLIGALLAIQGASFVA-------------------SSQISASLLEETWN-LV   36
Hydropsyche_angustipennis          1  ----MAILVFLSALLFIQAA---S-------------------AHCNTAGLVQATWG-LI   33
Parapsyche_elsis                   1  ----MAILVLLSALFFFQAA---A-------------------AHCNT-GLIEATWG-LI   32
Stenopsyche_tienmushanensis        1  ----MAILVFLSALLVFQAA---T-------------------ACNVPGGLLQAAWG-LI   33
Stenopsyche_marmorata              1  ----MAILVFLSALLVFQAA---T-------------------ACNVPGGLLQAAWG-LI   33
Rhyacophila_obliterata             1  ----MALLLLTAAFLATQGI---A-------------------SAAIQPALIEATWR-LV   33
Hydropysche_tenuis                 1  ----MAILVFLSALLFIQAA---S-------------------AHCNTAGLVQATWG-LI   33
                                          *         .

P21828      FIBL_BOMMO            51  DDTDKSIAILNVQEILKDMASQGDYASQASAVAQTAGIIAHLSAGIPGDACAAANVINSY  110
Q26427      FIBL_GALME            53  DGGDTNIYILTIQQILNDLADQPDGLSQSLAVTQAVAALGELATGVPGNSCEAAAVIDAY  112
Q9BLL5      Q9BLL5_PAPXU          52  DGGDTNIYILTIRQILNDLANQSDSKSQALAVGQAIAILGELAYGNPGDACAAAELVNAY  111
A0A6J2JLC8  A0A6J2JLC8_BOMMA      51  DDTDKSIAILNVQEILKDMASQGDYASQASAVAQTAGIIAHLSAGIPGDACAAANVINSY  110
A0A7I0ZNV9  A0A7I0ZNV9_DANPL      57  DGADIPLYAQMMIQSVNDQANSGDAASQASAVVLAINAIAELANGIPGDACESAALVNAY  116
Q9BLL7      Q9BLL7_9NEOP          54  DGGDNNIYILNAMQLMNDFANSGDSYSQARALAQTIATAIDLSSGIPGDACASADVANAY  113
A0A891XIJ7  A0A891XIJ7_TINBI      54  GGDKKTVFILSIEHVISDLLRQPDSYSQAMGLSNAVALVGELAPSAPGDACGSADMVNAY  113
Q14UU5      Q14UU5_YPOEV          53  DGAEQNIYMLTNQQIVNDMANSGDPTTQALALGQAINLVGEA-VGSTGDACAYANLANAY  111
A0A0N1PIW2  A0A0N1PIW2_PAPMA      52  DDGDSNIYILTIRQILNDLANQADPKSRALAVGQAIALLGELVYGSPGDACIAAELINAY  111
A0A3S2NFE9  A0A3S2NFE9_CHISP      54  DGGDSNVYTLTLLEGVHDLANQQDRNSQSLAIAQTIAILGELATGVPGQSCEAAAVINAF  113
A0A2A4JXS4  A0A2A4JXS4_HELVI      53  DGGDTVPYVLNFQQVVDDLANQGDKSSQALAAGQVIAILGSLSTGIPGDACAPANFIDSY  112
A0A821XWV1  A0A821XWV1_9NEOP      54  DGGSIPLYAQMLLQILNDLANSPNPVSKATAVIQTIAALGELSHGTSGDSCEAAGLINAY  113
A0A8B8HT70  A0A8B8HT70_9NEOP      54  DGGDTPLYAQMIIQSINDMADTSDTDSWANAVIQTIATVGELANGVPGDACEAAALINAY  113
D0EWD8      D0EWD8_CORCP          53  DGGDTNIYILTIQQILNDLANQPDSLSQNLAVVQTVAALGELATGAPGDSCEAAALVDAY  112
J9XSZ1      J9XSZ1_HARDE          53  DGGDQNIYILTIEQILNDLANQPDSLSQALAVGQTIAVLGELANGVPGDSCEAAALVNAY  112
V5UTC1      V5UTC1_OSTFU          53  DGGDTNIYILTIQQIINDLSNQPDPRSQALAVAQAIAVVGELASGIPGDACDASAFVNAY  112
A0A7E5W904  A0A7E5W904_TRINI      43  DGGDLTFNILTFEAIIKDLVSLPDPRSQALGFGQAIALVGSLATGIPGDACAAAQFIDNY  102
A0A6J1NAW1  A0A6J1NAW1_BICAN      53  DGGDVPLYLQILMTSVNDIAAV----NQAAAAIQTIVILGETAPGIPGDACEAAALINAS  108
A0A5B2VHG3  A0A5B2VHG3_9BACT      51  DDTDKSIAILNVQEILKDMASQGDYASQASAVAQTAGIIAHLSAGIPGDACAAANVINSY  110
A0A835GRS5  A0A835GRS5_SPOEX      53  NQGEASAYLLTFEQIIKDLGNDSNKVSQALAAAQAIGYLGAVSSGVPGDSCAAADLINAV  112
D7PBV9      D7PBV9_9NEOP          52  DGASVPIHALTLHQATLDLADQPDGVSQSIAAGQTLGILGELTSPVAGDTCGYSQLVDSY  111
A0A7U3TKS2  A0A7U3TKS2_PLUXY      53  DRGELNVYTLTAVNVLGDLAGAPDSYSQALAVGQTINLLAEL-TGPASDGCEYTNIINEA  111
Limnephilus_decipienns            37  EQGEVEPYVLLLKDEVVSTG-------GVYGLGATLTGVGELAWPRPASGCGHSKLINAN   89
Limnephilus_lunatus               37  EQGEVEPYVLLLKDEVVSTG-------GVYGLGATLTGVGELAWPRPASGCGHSKLINAN   89
Limnephilus_rhombicus             37  EQGEVEPYVLLLKDEVVSTG-------GVYGLGATLTGVGELAWPRPASGCGHSKLINAN   89
Limnephilus_marmoratus            37  EQGEVEPYVLLLKDEVVSTA-------GVYGLGATLTGVGELAWPRPASGCGHSKLINAN   89
Hesperophylax_occidentalis        37  DQGEVEPFTLLLKKEVVATG-------GVYGLGATLTGVGELAWPRPASGCGHSKLINAN   89
Hydropsyche_angustipennis         34  EDGEIEPFSLVLRDSILAIENDNXPTSQLYALGATLTAVSELSWVRPSSACAYANLINAN   93
Parapsyche_elsis                  33  EDGEIEPFSLVLRNNLLETANT-GRSGEIYALGATFSAVSELSWVRPASACAYANLINAN   91
Stenopsyche_tienmushanensis       34  EDGEIEPFALVLRNDILSN---SGLDGGLYALGATFTAVSELSWVRPASACAHANLINAN   90
Stenopsyche_marmorata             34  EDGEIEPFALVLRNDILSN---SGSDGGLYALGATFTAVSELSWVRPASACAHANLINAN   90
Rhyacophila_obliterata            34  EDGEIPPFALLLRDELIAEAGPSXSTXELYALGATFTAVGELAWPRAASGCGHSKLINAC   93
Hydropysche_tenuis                34  EDGEIEPFSLVLRDSIVAGENENXSNSQLYALGATFTAVSELSWVRPSSACAYANLINAN   93
                                         .                          .   .            .. *  : . :

P21828      FIBL_BOMMO           111  TDGVR-SGNFAGFRQSLGPFFGHVGQNLNLINQLVINPGQLRYSVGPALGCAGGGRIYDF  169
Q26427      FIBL_GALME           113  ANSVR-TGDNSALSIAVANYINRLSSNIGLISQLASNPDSLRYSSGPAGNCAGGGRSYQF  171
Q9BLL5      Q9BLL5_PAPXU         112  T-----SGNKAATRAALGNFIQSLVANIDSLVQLILNPNSVRYSSGKRGNCVGGGRSYNF  166
A0A6J2JLC8  A0A6J2JLC8_BOMMA     111  TDGVR-SGNFAGFRQSLGPFFGHVGQNLNLINQLVTNPGQLRYSVGPALGCAGGGRIYDF  169
A0A7I0ZNV9  A0A7I0ZNV9_DANPL     117  AYAVS-TGNSAGLRSALVKYVQRINANIDAIVRLVNSPDSVRYSSGPRGNCIGGGRSYQF  175
Q9BLL7      Q9BLL7_9NEOP         114  SAAVR-SGNPSGFRSALNRYIKYIASNLDSIVRIANNPNSGRYSVGPSGGCSGGGRSYDF  172
A0A891XIJ7  A0A891XIJ7_TINBI     114  A-----SGNQGAIRQAVSNYVSVINTNIDTIAKLAVNPSSLRYAVGPSGNCPGGGRSYQF  168
Q14UU5      Q14UU5_YPOEV         112  A-----SGNAAAVSQALSGYVNRLNANINAVARLAVDPTAAGSIVGSSGGCAGGGRSYQF  166
A0A0N1PIW2  A0A0N1PIW2_PAPMA     112  T-----SGNKAATSAALVNFIQSLVANIDSFVQLILNPNSVRYSSGRRGNCVGGGRNYNF  166
A0A3S2NFE9  A0A3S2NFE9_CHISP     114  AGSVR-TGNKSGLRPAVMNYLARLANNIDLIVGLVNNPDSLRSAVGPRGNCAGGGRRYEF  172
A0A2A4JXS4  A0A2A4JXS4_HELVI     113  VASLR-NGNS--VAPALGKFVGAIKQHIDQIVQYLNNPNQLKNAVGPRGNCNGGGRNYQF  169
A0A821XWV1  A0A821XWV1_9NEOP     114  AYSAK-SGNKSGLRQAIANFVARLSNNIDTIAQLIVNPNAVRYSVGPRGSCIGGGRTYQF  172
A0A8B8HT70  A0A8B8HT70_9NEOP     114  AYSVS-TGNSAGLRAALPNFVQRIAGYVDTIVQLVVNPNGVRYSSGPRGKCLGGGRSYQF  172
D0EWD8      D0EWD8_CORCP         113  ASAVR-TGNSGALAAAAGNYINRLLSSVNNIVQLANNPNAVRYTSGPSGSCSNGGRSYQF  171
J9XSZ1      J9XSZ1_HARDE         113  ANGVR-SGNFAGVRGALNNFLGRLASNIDLIAQVAANPNALRFSSGPKGNCAGGGRTYQF  171
V5UTC1      V5UTC1_OSTFU         113  ANAVR-SGNNAGLRAALNRYLGRLAADIDRIAQLAVNPNALRSAVGPRGNCAGGGRTYQF  171
A0A7E5W904  A0A7E5W904_TRINI     103  VNGVR-DGQ------SVFNFVNSLASQIDLVAQISSDSNALRFATGPRGNCNGGGRNYQF  155
A0A6J1NAW1  A0A6J1NAW1_BICAN     109  -------ANNAGLRPSLVSFIQRIAGYIDTIVRLVNNPNSVRYSSGSRGNCLGGGRSCQF  161
A0A5B2VHG3  A0A5B2VHG3_9BACT     111  TDGVR-SGNFAGFRQSLGPFFGHVGQNLNLINQLVINPGQLRYSVGPALGCAGGGRIYDF  169
A0A835GRS5  A0A835GRS5_SPOEX     113  VAASR-SGNNQAVRSATAKFVSYLSSLIDNIVELSVNSESMRFAVGPRGNCPAGGRNYQF  171
D7PBV9      D7PBV9_9NEOP         112  NNYHT-TGNRADLNNAIDSYVGSLNRAVDQLVLLRTNPAALRNQAAVVHSCSGGGRSYGH  170
A0A7U3TKS2  A0A7U3TKS2_PLUXY     112  V-----SGNSGA---AQRAYANRLARNIDTIVKFAQNPNAARLAASRNGSCAGGARGFNL  163
Limnephilus_decipienns            90  VALNDGTLAWGELEDAVDSYAVVLAQAVDNLRILGLNCIIPAPWPTLENSCGDWGRIYDF  149
Limnephilus_lunatus               90  VALNDGTLAWGELEDAVDSYAVVLAQAVDNLRILGLNCIIPAPWPTLENSCGDWGRIYEF  149
Limnephilus_rhombicus             90  VALNDGTLAWGELEDAVDSYAVVLAQAVDNLRILGLNCIIPAPWPTLENSCGDWGRIYDF  149
Limnephilus_marmoratus            90  VALNDGTLAWGELEDAVDSYAVVLAQAVDNLRILGLNCIIPAPWPTLENSCGDWGRIYDF  149
Hesperophylax_occidentalis        90  VALNDGTLAWGELEDAVDSYAVVLAQAVDNLRILGLSCIIPAPWPTLENSCGDWGRIYDF  149
Hydropsyche_angustipennis         94  VGLANHNLGRAALSSAIDGYAQVLAQAAENIRILGQCCVLPSPWPVLDNCCGDYGRIYDF  153
Parapsyche_elsis                  92  VHLGGGSLSRASLSNAIDGYAVSLAQAVENLRILGRCCVLPSPWPVLDNCCGDYGRIYDF  151
Stenopsyche_tienmushanensis       91  VNLARHSLGRDALSAAIDGYAVVLAQAAENFRLLGQPCVLPSPWPTLDNCCGDYGRIYQF  150
Stenopsyche_marmorata             91  VNLARHSLGRDALSAAIDGYAVVLAQAAENFRLLGQTCVLPSPWPTLDNCCGDYGRIYQF  150
Rhyacophila_obliterata            94  VGLNDGSTSYSELSDAIDSYAVVLSQAVDNLRILGYCCIVPAPWPPMDNSCHDYGRIYSF  153
Hydropysche_tenuis                94  VDLGNHNLGRAALSSAIDGYAQVLAQAAENIRILGQCCVLPSPWPVLDNCCGDYGRIYDF  153
                                                         :   :   :      .                   *   .*
```

```
P21828      FIBL_BOMMO                  170  EAAWDAILASS----DSSFLNEEYCIVKRLYNSRNSQSNNIAAYITAHLLPPVAQVFHQS  225
Q26427      FIBL_GALME                  172  EAAWDAVLNNAN-PYQIGLINEEYCAARRLYNAFNSRSNNVGAAITAGAVVAQTQAAQII  230
Q9BLL5      Q9BLL5_PAPXU                167  EAAWDSVLSEAD-PFLSSLYNEEYCAAKRLYNAFNIRSNNVGAAVTAASLSPVTKVVQRA  225
A0A6J2JLC8  A0A6J2JLC8_BOMMA            170  EAAWDAILASS----DSGFLNEEYCIVKRLYNSRNSQSNNIAAYITAHLLPPVAQVFHQS  225
A0A7I0ZNV9  A0A7I0ZNV9_DANPL            176  EEAWDDILNNAN-PFQIGLLNEEYCAAKRMYNAPSVRSNNLAATVTASLSRYVNQIGQNA  234
Q9BLL7      Q9BLL7_9NEOP                173  ESVWQSVLAGS----SSSLDYEGYCVAKRLYSAFNVRSNNIGAAITATSIPQVINVFEAV  228
A0A891XIJ7  A0A891XIJ7_TINBI            169  EAAWDRILESSN-PYQIGLVNEEYCSARRLYTANSVQSNNVGAAISASSTAPITQVFAQS  227
Q14UU5      Q14UU5_YPOEV                167  EQVWDSVLANAN-AYTIGLLNEQYCMARRLYASYNPQNNNVAAALSASAIPEVRQILSSV  225
A0A0N1PIW2  A0A0N1PIW2_PAPMA            167  EGAWDSVLSEAD-PFLSSLYNEEYCAAKRLYNAFNIRSNNVGAAVTAASLSPVTEVVQKA  225
A0A3S2NFE9  A0A3S2NFE9_CHISP            173  EAVWDAVLNSAN-INTVGLLNEQYCSSKRLYSAFNIRTNNVGAAATAAAVPQVTRAVGQA  231
A0A2A4JXS4  A0A2A4JXS4_HELVI            170  EAAWDAILSNAS-GASAGLVNEQYCAAKRLYSAFGLQSSSLGAYVTAVSLPPVNRVLQDA  228
A0A821XWV1  A0A821XWV1_9NEOP            173  EESWDAILSSAA-PAQSALLNEEYCVARRLYNAFNIRSNNVAAGVSAAAIPQVIKVAQYA  231
A0A8B8HT70  A0A8B8HT70_9NEOP            173  EEAWDGILDKAN-AYQIGLLNEEYCAAKRLYNAFNIRSNNVAAGVTAVTTRPVNQLVQLA  231
D0EWD8      D0EWD8_CORCP                172  EAAWDAVLSSAN-AYQIGLINEEYCAAKRLYSAFNTRSNNVGAAITAATVATQAGIADHL  230
J9XSZ1      J9XSZ1_HARDE                172  EAAWDAVLSSAN-AYQIGLINEEYCAAKRLYSAVNIRSNNVGAAVSAAAVAPVTQAVQGA  230
V5UTC1      V5UTC1_OSTFU                172  EAAWAAINNG-----SNGLINEEYCAAKRLYSAFNARSNNVGAAVSAAAAAPVARAVQQA  226
A0A7E5W904  A0A7E5W904_TRINI            156  EAAWDLIFDSAT-PSQFNYINEQYCTAKRLKSSVNVNSNNAAAVITAVSLPVVNNILLNS  214
A0A6J1NAW1  A0A6J1NAW1_BICAN            162  EAAWDRILRESS-PYDFGLLNEEYCAAKRLSTAWNLRSNNVATAITAATIPEVREVVRLG  220
A0A5B2VHG3  A0A5B2VHG3_9BACT            170  EAAWDAILASS----DSSFLNEEYCIVKRLYNSRNSQSNNIAAYITAHLLPPVAQVFHQS  225
A0A835GRS5  A0A835GRS5_SPOEX            172  EAAWDSILASAP-ASSVHLVNEQYCAAKRLYKGFSVVSNNAGAILTAVSLPSVNLAFQNV  230
D7PBV9      D7PBV9_9NEOP                171  DKVWDLALSNAGSAAHADLINEQQCSSRRLYGAWDRRSNSVAAAAAAVTSPGHQVVRGA  230
A0A7U3TKS2  A0A7U3TKS2_PLUXY            164  EAAWDSILSNA---SQIGLLNEQYCTVKRLYGATSASSNSAAALTAASLAPSAYVNEAA  220
Limnephilus_decipienns                  150  ESSWSLSKVN----------KGVVCAARRLYTSFGARANNVGAAATSAATDAATSIISEI  199
Limnephilus_lunatus                     150  ESSWSLSNVN----------NGVVCAARRLYTSFGARANNVGAAATSAATDAATSIISEI  199
Limnephilus_rhombicus                   150  ESSWGLSKVN----------KGVVCAARRLYTSFGARANNVGAAATSAATDAAVSIISEI  199
Limnephilus_marmoratus                  150  ESSWGLSKVN----------KGVVCAARRLYTSFGARANNVGAAATSAATDAAISIITEI  199
Hesperophylax_occidentalis              150  ENSWDLSNVN----------NGVVCAARRLYTAFGARANNVGAAATSAATDAAITIISDV  199
Hydropsyche_angustipennis               154  ENSWSLATGCNS--------EGPRCAARDLYLALNARSNNVGAAATSAATTPALSIFKRI  205
Parapsyche_elsis                        152  EDSWSLATSFSS--------SKARCAARDLYTALNARSNNVGAAATSAATTPALSILKCI  203
Stenopsyche_tienmushanensis             151  EESWDLANSA-S--------SGARCAARDLYTSFGARANNVGAAATSAATSPALAIFKGI  201
Stenopsyche_marmorata                   151  EESWDLANSA-S--------SVARCAARDLYTSFGARANNVGAAATSAATSPALAIFKGI  201
Rhyacophila_obliterata                  154  EDSWDLAKGA-G--------SKARCIARRLYTSFGARLNNIGAAATSAATIAAREILEQI  204
Hydropysche_tenuis                      154  ENSWGLVKGFSS--------NGPSCAARDLYLALNARSNNVGAAATSAATSPAIGIIKRI  205
                                             :  *                    *  : :  . .   .. .:  ::

P21828      FIBL_BOMMO                  226  AGSI--TDLLRGVGNG-----------NDATGLVANAQRYIAQAASQVHV-------  262
Q26427      FIBL_GALME                  231  LPSL--VNVLSAVAAG-----------GNVAGAAAQAGQALANAAANVQL-------  267
Q9BLL5      Q9BLL5_PAPXU                226  YSTL--ANLLRAIGNN-----------GNVIGAAAAAKVELLRSL------------  257
A0A6J2JLC8  A0A6J2JLC8_BOMMA            226  AGSI--TDLLRGVGNG-----------NDATGLVANAQRYIAQAASQVHV-------  262
A0A7I0ZNV9  A0A7I0ZNV9_DANPL            235  LGPL--AEFLRAAAIG-----------GNLNVSAGNAKAALSRALASVQY-------  271
Q9BLL7      Q9BLL7_9NEOP                229  LGPA--STFLRTIANG-----------GNAAQAAGKLRSALVNAASRT---------  263
A0A891XIJ7  A0A891XIJ7_TINBI            228  IGPV--SNFLRAAISG-----------QNVQGAAQAAKAAISNAARYI---------  262
Q14UU5      Q14UU5_YPOEV                226  AAPL--ANLMRVVASG-----------GNPAQAAASAQQALAQAAARA---------  260
A0A0N1PIW2  A0A0N1PIW2_PAPMA            226  YGSL--TNLLRAIGNN-----------GNVIGAAAAAKVELLRSV------------  257
A0A3S2NFE9  A0A3S2NFE9_CHISP            232  LNAV--VPFLKVVANG-----------GNPAGLAAGAKQALLRAGSSVPL-------  268
A0A2A4JXS4  A0A2A4JXS4_HELVI            229  LEPV--AKFVASLSSG------------NYAAAAAGAKNALDNALRY----------  261
A0A821XWV1  A0A821XWV1_9NEOP            232  LQPL--AQFLRAVASG-----------ENPLQPAAAAKAGLNQAVNKIKL-------  268
A0A8B8HT70  A0A8B8HT70_9NEOP            232  LPQLAQVSFLRVVANG-----------GNPAQAGANAKNALLQSLPKIQY-------  270
D0EWD8      D0EWD8_CORCP                231  IPSL--TSLLSSVASG-----------GNVASAAAQVRTAVSSGATKIRL-------  267
J9XSZ1      J9XSZ1_HARDE                231  LGPL--ANFLRAVANG-----------ANAASVAGAAKSALLQAGGRVQL-------  267
V5UTC1      V5UTC1_OSTFU                227  LNPL--SNFLRVVASG-----------ANPSSQAAAAKAALLRAGASIQL-------  263
A0A7E5W904  A0A7E5W904_TRINI            215  LNEI--TAFAGAVGQG------------NARAAAGAAKQALLRAASSY---------  248
A0A6J1NAW1  A0A6J1NAW1_BICAN            221  LPEV--GEFLRVLSYG-----------GNPGPAGQAAKAALLQRVSQVKC-------  257
A0A5B2VHG3  A0A5B2VHG3_9BACT            226  AGSI--TDLLRGVGNG-----------NDATGLVANAQRYIAQAASQVHV-------  262
A0A835GRS5  A0A835GRS5_SPOEX            231  LPQA--ANFLAALPSG------------NAGSAASALKQALYNSV------------  261
D7PBV9      D7PBV9_9NEOP                231  LPQI--ASFLNTAVAG-----------GNTAAAGQALKQALTQSSALVKSKY-----  269
A0A7U3TKS2  A0A7U3TKS2_PLUXY            221  LGPL--ANFLRSVASG-----------ANPAQAGLAAQRAIRSAV------------  252
Limnephilus_decipienns                  200  EDEL--VSYLEAVVSKSAGPK-----QKLLRTLAGSLKASIFRASGNAKSGLRSRCH  249
Limnephilus_lunatus                     200  EDEL--VSYLEAVVSKSAGPK-----QKLLRTLAGSLKASIFRASGNAKSGLRSRCH  249
Limnephilus_rhombicus                   200  EDEL--VSYLEAVVSKSAGPK-----QKLLRTLAGSLKASIFRASGNAKGGLRSRCH  249
Limnephilus_marmoratus                  200  DDEL--VSYLEAVVSKSAGPK-----DKKLRTLAGSLKASIFRASANAKGGLRSRCH  249
Hesperophylax_occidentalis              200  EDEL--VSYLEAVLSKSAGPGXCKSKQKQLRTLAGSLKASIFRASGIAKNGLRSRCH  254
Hydropsyche_angustipennis               206  KGEI--SSLLSLATAP--KSSGCATRKKDLRTAAGVLKQAIYNAADDVKSSLYSSCV  258
Parapsyche_elsis                        204  EGDL--IKFLEVA--T--SKSGCASRSKQLRTLGGILKSSIFSAADEAKSSLYHSCA  254
Stenopsyche_tienmushanensis             202  EGEL--VSVLKAATSK--------DCSRNLRTETGILKAAIFRAADEAKNSLYCRCV  248
Stenopsyche_marmorata                   202  EGEL--ISLLKAATSK--------DCSRNLRTETGLLKAAIFRAADEAKNSLYCRCV  248
Rhyacophila_obliterata                  205  ENDL--ITYLNTVVKSASGSWQCAQKKKNMLTLGGYLKSAIWKAASVTKRNLS----  255
Hydropysche_tenuis                      206  RGEL--SSLLSLATAP--KSSGCCTRKKDLRTAAGVLKQAIYNAADDVKNIP-----  253
                                                                                     :
```

**Supplemental Figure S1.** Multiple sequence alignment of 22 lepidopteran (blue) and 11 trichopteran (black) L-fibroin sequences. * identical, : strongly conserved, . less strongly conserved.

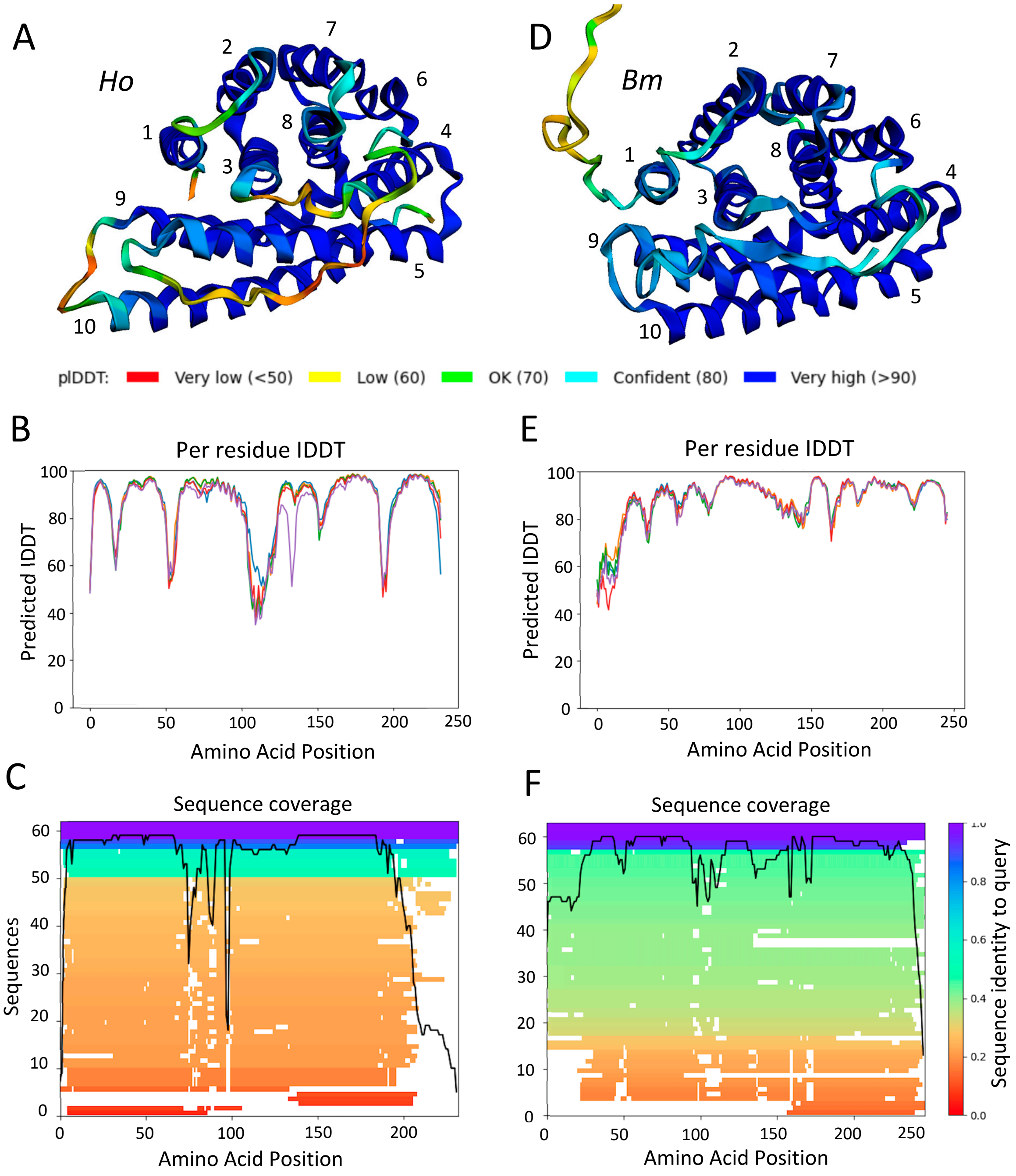


**Supplemental Figure S2.** Colabfold confidence metric scores and sequence coverage for *Ho* (A-C) and *Bm* (D-F) L-fibroin predicted structures.

**Supplementary Methods:**

Five of the Trichoptera L-fibroin sequences SFig. 1 were retrieved UniProtKB: (*Stenopsyche marmorata* LC057252.1,[1] *Rhyacophila obliterata* AB354690.1,[2] *Limnephilus decipienns* AB214510.1, [3] *Hydropsyche angustipennis* AB354593.1,[3] and *Hesperophylax occidentalis*: KM384738.1.[6] Two other sequences were obtained from previous publications (*Stenopsyche tienmushanensis* [4], *Hydropsyche tenuis* [5],). L-Fibroin sequences of four addditional species are newly reported in this study and were mined from published genomes (*Parapsyche elsis*: GCA_022651745.1 [7], *Limnephilus marmoratus:* GCA_917880885.1, *Limnephilus rhombicus:* GCA_929108145.1, *Limnephilus lunatus:* GCA_917563855.2 [8]). *L-fibroins* were extracted from the assemblies by using tBLASTn to identify conserved N- and C-terminal amino acids of closely related *Limnephilus decipienns* AB214509 [3] or *Hydropsyche angustipenni*s [3] in Geneious Prime 2022.1.1. Hits were extracted with 10,000 bps of flanking sequence using the sequence view "extract" in Geneious. For *Parapsyche elsis*, we annotated this region using Augustus v.3.3.2. [9]. The three *Limnephilus* species were annotated manually using the annotation of previous published *Limnephilus decipienns* AB214510.1 [3]. Protein coding nucleotide sequences were translated in Geneious. Newly reported L-fibroin sequences can be found on figshare: https://doi.org/10.6084/m9.figshare.20240847.v1

**Supplementary Material References:**


1. Bai,X., et al., *Molecular cloning, gene expression analysis, and recombinant protein expression of novel silk proteins from larvae of a retreat-maker caddisfly, Stenopsyche marmorata.* Biochem. Biophys. Res. Commun. 2015. **464**(3): p. 814-819.

2. Yonemura, N., et al., *Conservation of silk genes in Trichoptera and Lepidoptera.* J Mol Evol, 2009. **68**(6): p. 641-53.

3. Yonemura, N., et al. *Protein composition of silk filaments spun under water by caddisfly larvae.* Biomacromolecules 2006. **7**(12): p. 3370-3378.

4. Luo, S., et al., *The genome of an underwater architect, the caddisfly Stenopsyche tienmushanensis Hwang (Insecta: Trichoptera),* GigaScience, 2018. **7**(12): p. giy143, https://doi.org/10.1093/gigascience/giy143

5. Heckenhauer, J., et al., *Annotated Draft Genomes of Two Caddisfly Species Plectrocnemia conspersa CURTIS and Hydropsyche tenuis NAVAS (Insecta: Trichoptera).* Genome Biol Evol, 2019. **11**(12): p. 3445-3451.

6. Wang, C.S., et al., *Peroxinectin catalyzed dityrosine crosslinking in the adhesive underwater silk of a casemaker caddisfly larvae, Hysperophylax occidentalis.* Insect Biochem. Mol. Biol. 54, 2014. p. 69-79

7. Heckenhauer, J., et al., *Genome size evolution in the diverse insect order Trichoptera*. *GigaScience* 2022. 11: p. giac011, https://doi.org/10.1093/gigascience/giac011

8. WELLCOME SANGER INSTITUTE, https://www.sanger.ac.uk/collaboration/darwin-tree-of-life-project/

9. Stanke, M., et al., U*sing native and syntenically mapped cDNA alignments to improve de novo gene finding*. Bioinformatics, 2008. **24**: p. 637–644.
